# A Statistical Error in the Estimation of the Recommended Dietary Allowance for Vitamin D

**DOI:** 10.3390/nu6104472

**Published:** 2014-10-20

**Authors:** Paul J. Veugelers, John Paul Ekwaru

**Affiliations:** School of Public Health, University of Alberta, 350 University Terrace, Edmonton, AB T6G 2T4, Canada

The Institute of Medicine (IOM) issues dietary recommendations on the request of the U.S. and Canadian governments. One of these recommendations is the Recommended Dietary Allowance (RDA). The RDA is the nutrient intake considered to be sufficient to meet the requirements of 97.5% of healthy individuals [1]. The RDA for vitamin D is 600 IU per day for individuals 1 to 70 years of age and is assumed to achieve serum 25-hydroxyvitamin D (25(OH)D) levels of 50 nmol/L or more in 97.5% of healthy individuals [1]. Serum 25(OH)D is the established proxy for vitamin D status and levels of 50 nmol/L or more have been shown to benefit bone health and to prevent disease and injury [1].

**Figure 1:**
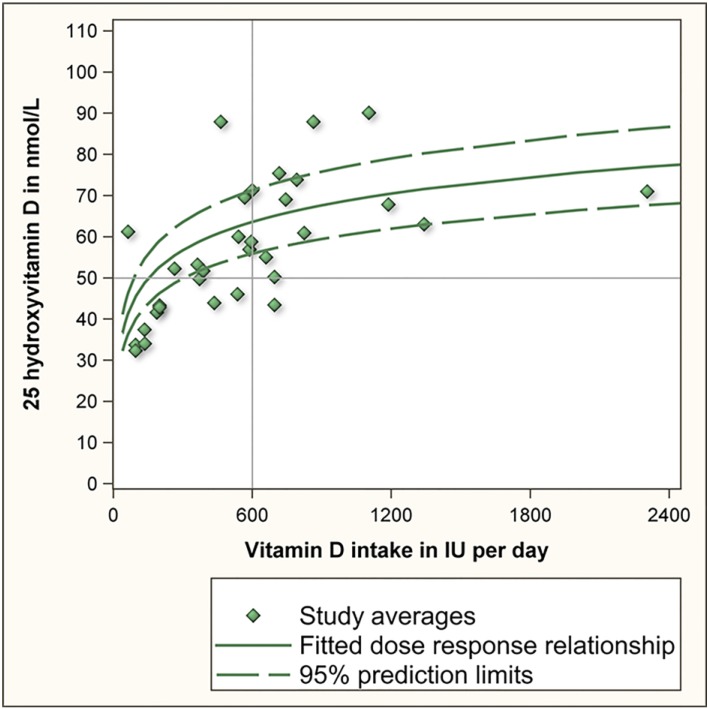
Dose response relationship of vitamin D intake and serum 25 hydroxyvitamin D.

The IOM based their RDA for vitamin D on an aggregation of 10 supplementation studies that were carried out during winter months and at locations with latitudes above the 50th parallel north to minimize the influence of cutaneous vitamin D synthesis [2,3,4,5,6,7,8,9,10,11]. As several of these 10 studies examined more than one supplementation dose, collectively they provided 32 study averages of serum 25(OH)D levels. These are replicated as the green diamonds in Figure 1. The IOM regressed the 32 study averages against vitamin D intake to yield the dose response relationship of vitamin D intake and serum 25(OH)D (green solid line in Figure 1). The IOM further calculated the lower and upper 95% confidence prediction interval based on the 32 study averages and the standard deviation of these 32 study averages (green dashed lines in Figure 1). On the basis of this, the IOM estimated that 600 IU of vitamin D would achieve an average 25(OH)D level of 63 nmol/L and a lower 95% confidence prediction limit (2.5 percentile) of 56 nmol/L. The latter value was rounded downwards to 50 nmol/L to accommodate uncertainty in the estimation [1]. This data point (600 IU vitamin D, 50 nmol/L) is the basis for the current RDA and for the IOM’s conclusion that an intake of 600 IU of vitamin D per day will achieve serum 25(OH)D levels of 50 nmol/L or more in 97.5% of individuals. This conclusion, however, is incorrect.

**Figure 2:**
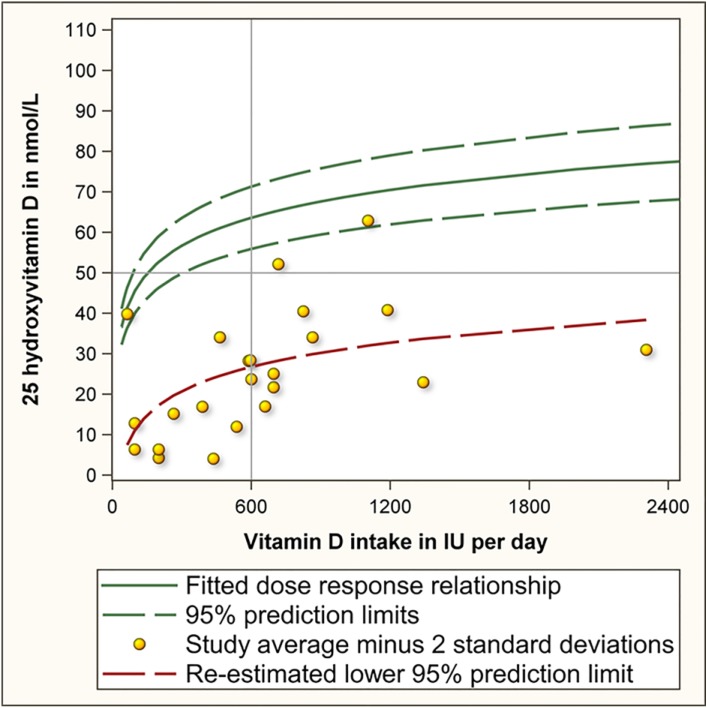
Dose response relationship of vitamin D intake and serum 25 hydroxyvitamin D.

The correct interpretation of the lower prediction limit is that 97.5% of study averages are predicted to have values exceeding this limit. This is essentially different from the IOM’s conclusion that 97.5% of individuals will have values exceeding the lower prediction limit. To illustrate the difference between the former and latter interpretation, we estimated how much vitamin D is needed to achieve that 97.5% of individuals achieve serum 25(OH)D values of 50 nmol/L or more. For this purpose we reviewed each of the 10 studies used by the IOM. Eight studies reported both the average and standard deviation [2,5,6,7,8,9,10,11]. These eight studies had examined a total of 23 supplementation doses [2,5,6,7,8,9,10,11]. For each of these 23 study averages we calculated the 2.5th percentile by subtracting 2 standard deviations from the average (depicted by yellow dots in Figure 2). Next, we regressed these 23 values against vitamin D intake to yield the lower prediction limit (red line in Figure 2). This regression line revealed that 600 IU of vitamin D per day achieves that 97.5% of individuals will have serum 25(OH)D values above 26.8 nmol/L rather than above 50 nmol/L which is currently assumed. It also estimated that 8895 IU of vitamin D per day may be needed to accomplish that 97.5% of individuals achieve serum 25(OH)D values of 50 nmol/L or more. As this dose is far beyond the range of studied doses, caution is warranted when interpreting this estimate. Regardless, the very high estimate illustrates that the dose is well in excess of the current RDA of 600 IU per day and the tolerable upper intake of 4000 IU per day [1].

The public health and clinical implications of the miscalculated RDA for vitamin D are serious. With the current recommendation of 600 IU, bone health objectives and disease and injury prevention targets will not be met. This became apparent in two studies conducted in Canada where, because of the Northern latitude, cutaneous vitamin D synthesis is limited and where diets contribute an estimated 232 IU of vitamin D per day [12]. One study estimated that despite Vitamin D supplementation with 400 IU or more (including dietary intake that is a total intake of 632 IU or more) 10% of participants had values of less than 50 nmol/L [13]. The second study reported serum 25(OH)D levels of less than 50 nmol/L for 15% of participants who reported supplementation with vitamin D [14]. If the RDA had been adequate, these percentages should not have exceeded 2.5%. Herewith these studies show that the current public health target is not being met.

We recommend that the RDA for vitamin D be reconsidered to allow for appropriate public health and clinical decision-making.
